# Effect of ivermectin on *Toxocara canis* larvae *in vitro* and *in vivo* at different migratory stages

**DOI:** 10.7705/biomedica.7828

**Published:** 2026-03-02

**Authors:** Iman F. Abou-El-Naga, Suzanne M.F. El-Nassery, Mona M. El-Temsahy

**Affiliations:** 1 Medical Parasitology Department, Faculty of Medicine, Alexandria University, Alexandria Governorate, Egypt Alexandria University Faculty of Medicine Alexandria University Alexandria Governorate Egypt

**Keywords:** Toxocara canis, toxocariasis/pathology, ivermectin, microscopy, electron, scanning, Toxocara canis, toxocariasis/patología, ivermectina, microscopía electrónica de barrido

## Abstract

**Introduction.:**

Ivermectin has shown potent activity against various nematodes.

**Objective.:**

Evaluation of the efficacy of ivermectin against *Toxocara canis* larvae.

**Materials and methods.:**

*In vitro* assessment was conducted using larval migration inhibition test and scanning electron microscopy (SEM). *In vivo,* ivermectin was administered orally to mice at a single 0.2 mg/kg dose at 2 (group I), 7 (group II), or 15 days (group III) post-infection. Efficacy was evaluated through brain larval counts and histopathological examination of liver and brain.

**Results.:**

*In vitro,* ivermectin at concentrations of 10 pg/ml and 100 pg/ml inhibited larval migration by 8.5% and 87%, respectively. SEM showed cuticular damage after 24 and 48 hours with 100 pg/ml ivermectin. *In vivo,* brain larval counts significantly decreased in groups I and II (22.67 ± 5.41 and 37.17 ± 5.98), with no significant reduction in group III (130.5 ± 9.01). Histopathology of the liver at four weeks post-infection in groups I and II revealed numerous granulomas with degenerated larvae. The brain showed a marked reduction in larval presence and inflammation in groups I and II, while group III findings were similar to those of the control group.

**Conclusion.:**

Ivermectin is effective against *T. canis* larvae during early migratory stages but shows limited efficacy against late-stage larvae.

## Resumen

**Introducción.:**

La ivermectina ha demostrado una potente actividad contra diversos nematodos.

**Objetivo.:**

Evaluar la eficacia de la ivermectina contra las larvas de *Toxocara canis.*

**Materiales y métodos.:**

La evaluación *in vitro* se realizó mediante la prueba de inhibición de la migración larval y microscopía electrónica de barrido. *In vivo,* se administró ivermectina por vía oral a ratones en una dosis única de 0,2 mg/kg a los 2 (grupo I), 7 (grupo II) o 15 días (grupo III) después de la infección. La eficacia se evaluó mediante el recuento larval en cerebro, y los exámenes histopatológicos del hígado y cerebro.

**Resultados.:**

*In vitro,* la ivermectina en concentraciones de 10 pg/ml y 100 pg/ml inhibió la migración larval en el 8,5 y el 87 %, respectivamente. La microscopía electrónica de barrido mostró daño en la cutícula tras 24 y 48 horas con 100 pg/ml de ivermectina. *In vivo,* el recuento larval cerebral disminuyó significativamente en los grupos I y II (22,67 ± 5,41 y 37,17 ± 5,98), sin reducción significativa en el grupo III (130,5 ± 9,01). La histopatología hepática a las cuatro semanas después de la infección mostró numerosos granulomas con larvas degeneradas en los grupos I y II. El cerebro presentó una acentuada reducción de larvas e inflamación en los grupos I y II, mientras que los hallazgos del grupo III fueron similares a los del grupo control.

**Conclusión.:**

La ivermectina es eficaz contra las larvas de *T. canis* durante las etapas migratorias tempranas, pero muestra una eficacia limitada en las etapas larvales tardías.

Toxocariasis is a parasitic zoonosis caused primarily by *Toxocara canis,* an ascarid nematode of dogs and other canids. The disease is prevalent in both developed and developing countries, representing a major public health concern in tropical regions, where the main focus of infection occurs ^1^. Immature eggs are released into the environment with the feces of infected pups. Eggs reach maturity after the development of third-stage larvae ^2^. In young dogs, usually less than six months old, the worms complete their full life cycle, including a pulmonary larval phase, followed by maturation into adult worms in the small intestine.

However, in adult dogs, the larvae usually do not reach adulthood, but remain viable within various tissues. During pregnancy, these larvae are activated and migrate from the tissues to the fetuses, resulting in pups being born infected ^3^. In companion animal hosts, the pathology usually arises from entero-hepatic-pulmonary migration of juvenile worms, causing respiratory and intestinal symptoms ^4^. However, these symptoms are often mild or asymptomatic. Diarrhea may occur, particularly when there are co-infections with other pathogens ^5^. In cases of heavy infection, animals may suffer impaction of the small intestine, potentially leading to intestinal rupture and subsequent septic peritonitis ^3^.

Humans become infected mainly after ingestion of mature eggs or larvae in infected meat of animals acting as paratenic hosts ^6^. The eggs hatch in the small intestine and the larvae migrate throughout the body without further development. In humans, migrating larvae can lead to visceral or ocular manifestations that potentially lead to permanent vision loss ^7^. Neurotoxocariasis may also occur due to migration of larvae to the brain and spinal cord ^1^. Furthermore, toxocariasis can cause a non-specific febrile illness accompanied by anorexia, nausea and respiratory symptoms, often referred to as common toxocariasis, which usually presents as a self-limiting condition ^8^. The disease is often associated with eosinophilia, a notable characteristic of tissue-invasive helminths ^9^.

Currently, there is a lack of adequate anthelmintic drugs for the effective treatment of human toxocariasis. Unfortunately, the anthelmintics commonly used to treat intestinal helminthiasis are often less effective against tissue-dwelling parasites, such as those causing visceral toxocariasis. Albendazole has demonstrated superior efficacy compared to other drugs and has therefore become the preferred treatment for human toxocariasis ^10^, despite a cure rate of less than 60% ^11^.

Ivermectin is a macrocyclic lactone that exhibits a broad spectrum of anthelmintic activities and target multiple molecular sites in nematodes ^12^. It acts on glutamate-gated chloride channels, causing muscle paralysis and inhibition of larval motility ^13^. The drug also affects the release of the excretory and secretory products ^14^, the main source affecting the host immune responses ^15^. Ivermectin also enhances the adhesion of mononuclear cells and activated neutrophils to the nematode cuticle ^16^.

Adult *T. canis* worms in dogs can be effectively eliminated with a variety of anthelmintic drugs; however, none have been able to successfully eradicate the tissue-dwelling larval stage. Among these drugs, ivermectin has been shown to be effective against adult *T. canis* worms ^17^, although its efficacy against the larval stage has varied. Ivermectin is particularly effective against reactivated *T. canis* larvae and has been shown to significantly reduce vertical transmission when administered to pregnant dogs, resulting in a 90% reduction in infection rates among pups ^18^. However, the drug was not effective against non-reactivated somatic larvae in dogs ^19^^,^^20^.

Additionally, ivermectin demonstrated superior efficacy than albendazole and levamisole in the treatment of *Toxocara vitulorum* infections in buffalo calves ^21^.

Variable results have also been reported on the efficacy of ivermectin against *T. canis* larvae in paratenic hosts. Abo-Shehada and Herbert ^22^ found that the effects of ivermectin were comparable to those of levamisole, albendazole, and fenbendazole in mice. Fok and Kassai ^23^ demonstrated a moderate larvicidal potential of ivermectin, whereas Carrillo and Barriga ^24^ concluded that somatic *T. canis* larvae in mice are not eliminated by the drug.

In this study, we evaluated the *in vitro* effect of ivermectin on the third stage larvae of *T. canis. In vitro* testing is widely recognized in the veterinary field for assessing the efficacy of treatments against nematodes, offering a simple and cost-effective method for drug evaluation. The larval migration inhibition assay is commonly used to identify new anthelmintic compounds ^25^ or to detect anthelmintic resistance ^26^. A key advantage of these *in vitro* tests is that they enable direct contact between the tested compounds, and the parasite life stages, facilitating preliminary efficacy evaluations. This assay relies on the principle that *in vitro* drug exposure reduces the ability of nematodes to migrate across a barrier, such as a fine mesh. The resulting motor paralysis caused by ivermectin, together with the critical role of migration of *T. canis* larvae, influenced our selection of the larval migration inhibition assay as a suitable method to assess the effects of ivermectin on these larvae.

Following evidence of ivermectin activity *in vitro* studies, *in vivo* tests were conducted to validate these findings. One possible explanation for the variation in the efficacy of anthelmintic drugs against the larval stage of *T. canis* is that the susceptibility of *Toxocara* larvae may differ depending on their migratory stage within paratenic hosts ^22^. Therefore, the present study investigates the *in vitro* activity of ivermectin on *T. canis* larvae and the relative susceptibility of the larvae to the drug at different post-infection migratory stages in mice.

## Materials and methods

### Preparation of Toxocara canis larvae

Adult female *T. canis* worms were collected from the intestines of pups after anaesthetization by intravenous injection of 150 ml/kg of thiopental sodium ^27^. Subsequently, the worms were rinsed with sterile physiological saline solutions.

Eggs were then harvested from the vagina and the distal third of the uterus. They were cultured in 1% formalin following the method described by Abou-El-Naga ^2^. Larval development was monitored continuously over 30 days until full development of third stage larvae. Third-stage larvae usually develop after approximately three weeks of culture. However, by day 30, maximum infectivity was achieved ^28^^,^^29^. These larvae are well differentiated, characterized by well-developed lips, a granular intestinal region, and a distinct excretory pore. When larvae inside the eggs are exposed to light stimulation, they require 3 to 5 minutes of observation before any movement becomes noticeable. In contrast, earlier-stage larvae are highly sensitive to light and exhibit active movement immediately upon exposure to the microscope light during examination ^2^.

Fully mature eggs were used for infection of the animals and for extraction of viable larvae. Mature eggs were filtered through gauze, centrifuged and washed three times with sterile physiological saline solution. A stock solution of eggs in saline was prepared and stored at 4^°^C. A working solution (approximately 30,000 eggs) for mice infection was prepared with 800 eggs/0.2 ml. For larval extraction, the outer shell was removed from approximately 4,000 eggs. The concentrated eggs were incubated in 1% sodium hypochlorite solution at room temperature for 12 hours ^29^. Decorticated eggs were then washed with RPMI-1640 medium containing 1% (w/v) glucose and rotated for 5 minutes to induce hatching. Larvae were collected using Baermann's apparatus ^30^.

### In vitro motility assays

These were carried out using a larval migration inhibition assay as described by Wagland *et al.*^31^ and Evans *et al.*^32^, with some modifications. This method employs a migration system to physically separate motile larvae from non-motile ones using a filter mesh following preincubation, while they remain in continuous contact with substances that influence their motility. The mesh size is designed to permit the passage of active larvae and retain immotile larvae. The mesh size was selected based on the diameter of *T. canis* larvae, which varies between 18 and 20 urn ^33^.

Larvae were prepared at a concentration of 2,000 larvae/ml in RPMI-1640 (Lonza BioWhittaker, Basel, Switzerland) containing streptomycin (1 mg/ml), penicillin (1,000 units/ml), gentamicin (0.2 mg/ml) and amphotericin (2.5 mg/ ml). Petri dishes were prepared with 100 μl of larval suspension (each dish containing 200 larvae).

Larvae were preincubated with 750 μl of ivermectin (Ivomec, Merk Sharp & Dohme) at concentrations of 10 μg/ml and 100 μg/ml. Larvae without the drug served as a control. After two hours of pre-incubation at 37°C in 5% CO_2_, the larval suspension from each dish was incubated with the ivermectin solution and was transferred to the larval migration inhibition glass tubes. Each tube measured 35 mm in length and 7 mm in diameter, with one end fitted with a 10 mm nylon mesh disc (20 μm) (Sefar, Inc., Heiden, Switzerland).

Larval migration inhibition tubes were positioned into the wells of a tissue culture plate, with rubber rings around each tube to hold them in place at the edges of the well, ensuring that the bottom of the mesh was just above the base of the well. After two hours, the larval migration inhibition tubes were removed, and the number of larvae in each well was counted. Three experiments were performed for each drug concentration. Larvae were considered viable for the larval migration inhibition assay if more than 80% of control larvae in the culture tubes had migrated.

### Scanning electron microscopy

Approximately 800 larvae were used in total. Two hundred larvae were incubated, either with or without the drug, for 24 or 48 hours. Larvae were incubated with 100 μg/ml ivermectin at 37°C in a 5% CO_2_ incubator for 24 and 48 hours and processed for examination by scanning electron microscopy. This drug concentration was chosen as it was effective in larval migration inhibition assay. Larvae incubated with RPMI-1640 medium at 37°C in a 5% CO_2_ incubator for 24 and 48 hours served as control.

### In vivo studies

Thirty laboratory-bred, parasite-free male Swiss albino mice, 4 to 6 weeks old and weighing 20 to 25 grams, were used. The mice were assigned to five equal groups each of six mice. Twenty-four mice were inoculated orally, each with 800 mature eggs in 0.2 ml physiological saline using a syringe fitted with a blunt needle. Ivermectin was administered orally in a single dose of 0.2 mg/kg of body weight ^22^ at 2 days (group I), 7 days (group II) or 15 days (group III) post-infection. This scheme can detect the effect of the drug in the early migratory phase (groups I and II) and in the myotropic-neurotropic phase (group III). Group IV was the control, non-treated infected group. Six mice (group V) were the untreated non-infected control group. All mice were anaesthetized by intravenous injection of 150 ml/kg of thiopental sodium ^27^ and decapitated four weeks post-infection and subjected to the following *in vivo* studies.

*Brain larval count:* Half of the brain was squashed between two glass slides; larval motility was observed, and the larvae were counted under the light microscope. The total number of larvae in the entire brain was then calculated. The percentage reduction in brain larvae counts compared to the control infected untreated group was determined. The other half of the brain was processed for histopathological examination.

*Histopathological study:* Liver and brain sections were examined after hematoxylin and eosin staining.

### Statistical analysis

Data was entered and analyzed using the IBM SPSS™ software package (version 20.0; Armonk, NY, IBM Corp). Statistical analysis was performed using Welch's t-test, to compare each experimental group with the control infected untreated group. One-way ANOVA was employed to assess differences among all groups, followed by Tukey's post hoc test for pairwise comparisons. Statistical significance was set at p < 0.05. Error bars in the charts represent standard deviations.

The percentage of larvae inhibited from migration, as well as the percentage reduction in brain larvae counts, were calculated from the formula:




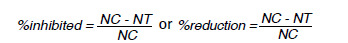




To determine the percentage of larvae inhibited from migration, NC represents the number of migrated larvae from the control tube and NT represents number of migrated larvae from the test tubes. To determine the mean percentage reduction in brain larval counts, NC represents the mean number of larvae detected in the infected control group and NT is the mean number of larvae detected in ivermectin-treated group at four weeks post-infection.

### Ethics committee permission

All animal studies were conducted in compliance with the regulations of the Ethics Committee of the Faculty of Medicine of Alexandria University, following Egyptian guidelines for animal experimentation (00012098).

## Results

The mean number of larvae recovered in the wells after incubation in RPMI-1640 was 195.3. The percentage of larvae inhibited from migration after incubation with ivermectin at a concentration of 10 μg/ml and 100 μg/ml was 8.5% and 87%, respectively.

**Figure 1:**
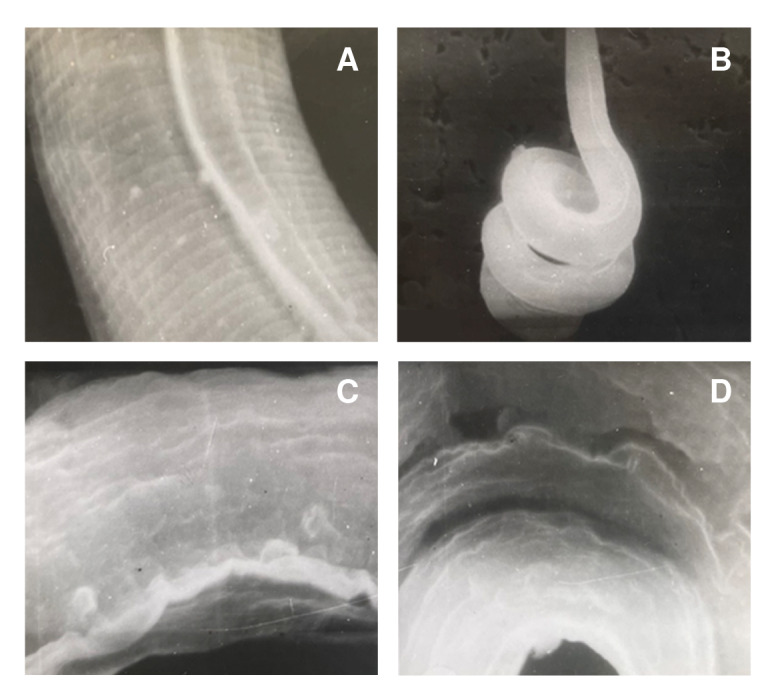
Scanning electron microscopy of *Toxocara canis* larvae. **A.** *Toxocara canis* larvae incubated in RPMI-1640 medium at 37°C in a 5% CO_2_ incubator for 24 and 48 hours, displaying prominent transverse striations and two lateral longitudinal alae (7,000X). **B.** The lateral alae extend from the anterior to posterior ends of the larvae (1,000X). **C.** Larvae after 24 hours of incubation with ivermectin, showing flattened transverse striations and wrinkled lateral alae (7,000X). **D.** Larvae after 48 hours of incubation with ivermectin, showing cuticular damage, characterized by deep grooves with irregular edges (1,000X).

Examination of *T. canis* larvae incubated in RPMI-1640 medium at 37°C in a 5% CO_2_ incubator for 24 and 48 hours, observed via scanning electron microscopy, revealed prominent transverse striations and two lateral longitudinal alae (figures 1a and 1b). After 24 hours of incubation with ivermectin at a concentration of 100 μg/ml, the transverse striations flattened and the lateral alae showed wrinkles (figure 1c). After 48 hours of incubation, cuticle damage was evident, characterized by deep grooves with irregular edges (figure 1d).

**Figure 2:**
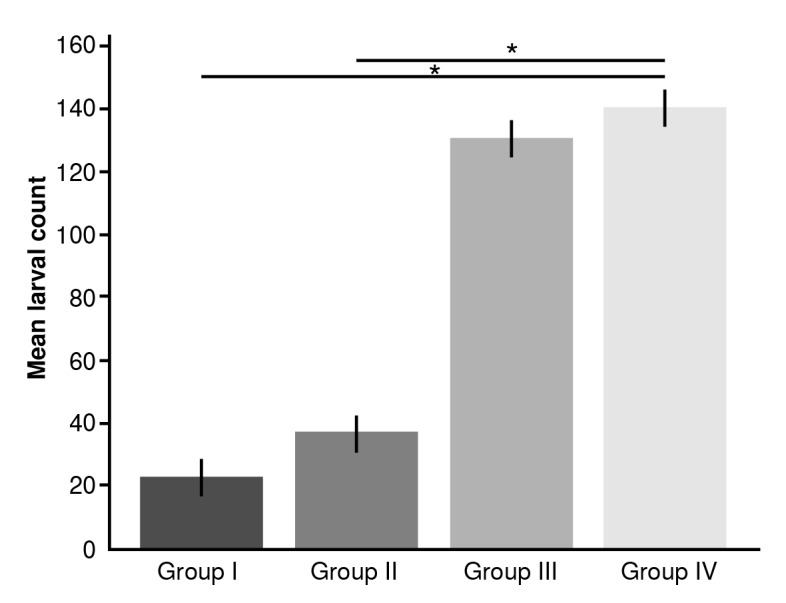
Mean brain larval count of all groups. Significant differences (*) with a p value < 0.05 were revealed between the count in group I and group II compared to the infected, untreated control group IV. Group III showed no significant differences compared to the control group.

The mean brain larval count of the infected, untreated control group (group IV) was 140.5 ± 8.43. In contrast, the mean brain larvae counts of the ivermectin-treated groups at two- and seven-days post-infection (group I and II) was 22.67 ± 5.41 and 37.17 ± 5.98, respectively, both significantly lower than that of the control group (p < 0.05). The larvae reduction percentages were 83.87% and 73.55%, respectively. However, when ivermectin was administered 15 days post-infection (group III), the larval count was 130.5 ± 9.01, with no significant difference from the control group (p > 0.05), with a larvae reduction percentage of 7.12% (figure 2).

There was a statistically significant difference between group I and group II, and between group II and group III (p < 0.05). Additionally, larvae in the brains of ivermectin-treated mice from groups I and II showed reduced motility during counting, compared to those in the control group.

**Figure 3:**
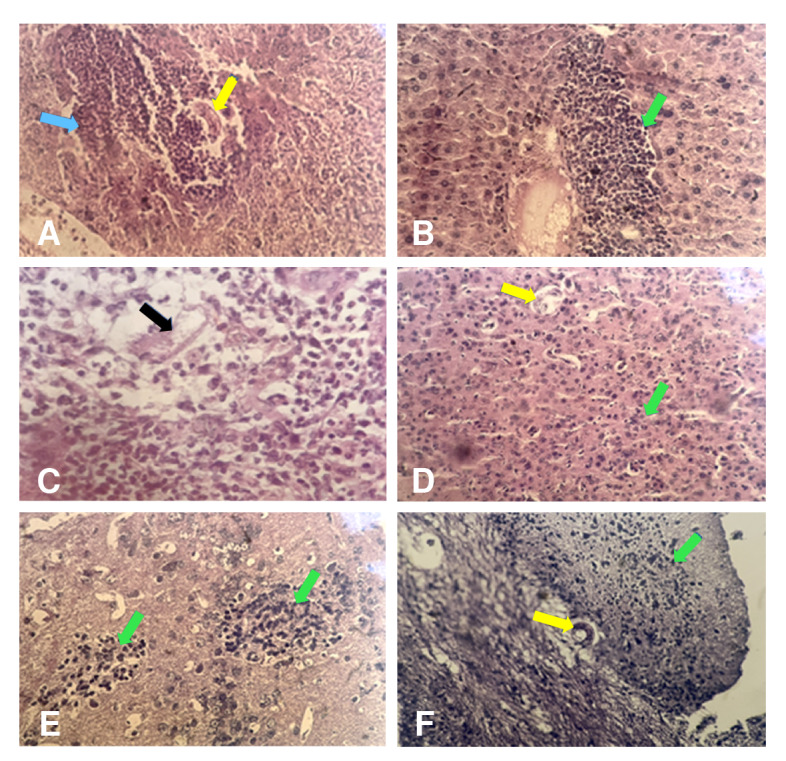
Histopathological examination of the liver and brain at four weeks post-infection. **A.** The liver of control-infected, untreated mice (group IV) shows granulomas (blue arrow) that frequently contain viable and intact larvae (yellow arrow), haematoxylin and eosin (40X). **B.** Marked cellular infiltration (green arrow) observed around several portal tracts in the liver of control-infected, untreated mice, haematoxylin and eosin (40X). **C.** A liver granuloma from mice in group I showing degenerated larvae (black arrow) after ivermectin treatment, haematoxylin and eosin (40X). **D.** The brain of control mice exhibits a high number of larvae (yellow arrow) accompanied by scattered inflammatory cells, haematoxylin and eosin (20X). **E.** Focal inflammatory reactions (green arrow) observed in control mice brain, haematoxylin and eosin (30X). **F.** The brain of mice in group I shows a reduced number of larvae (yellow arrow) and inflammatory reactions (green arrow) after ivermectin treatment, haematoxylin and eosin(30X).

Four weeks post-infection, the livers from infected, untreated control mice, exhibited granulomas composed predominantly of lymphocytes, plasma cells, eosinophils and macrophages, with viable, intact larvae frequently observed in the center of these granulomas (figure 3a). Additionally, a marked cellular infiltration was noted around several portal tracts (figure 3b). Histopathological examination of the liver from mice in groups I and II treated with ivermectin at 2 and 7 days post-infection, revealed numerous granulomas, some of which contained degenerated larvae (figure 3c), whereas the histopathological findings in group III were largely similar to those of the control group.

Histopathological examination of the brains of control mice at four weeks post-infection revealed many larvae accompanied by scattered and focal inflammatory cells (figures 3d and 3e). In contrast, treated groups I and II exhibited marked reductions in both the number of larvae and the extent of inflammatory reactions, with this reduction being particularly pronounced in group I (figure 3f). However, histopathological brain examination of mice treated with ivermectin at 15 days post-infection (group III) showed no notable differences compared to the control group.

## Discussion

The larvae migration inhibition assay used in this work was adapted to study the effect of ivermectin on third-stage *T. canis* larvae *in vitro.* The assay measures the number of larvae unable to actively migrate through a mesh after incubation with ivermectin. This assay was chosen as a biologically relevant and effective method to assess the impact of ivermectin on *T. canis* third-stage larvae, given the drug's paralytic effect on nematodes and the critical role of *T. canis* larval mobility within their hosts. There are several *in vitro* assays for evaluating drug effects, with the larval development assay being one of the most widely used ^34^^,^^35^. However, the distinct biology of *T. canis* larvae limits the applicability of this assay.

The larval migration inhibition assay described here allowed a high and consistent level of migration in untreated control larvae. A mesh size of 20 urn was chosen based on larval dimensions ^33^. The results obtained with ivermectin showed that the test is dependent on the concentration of the drug. Incubation of the larvae with 10 μg/ml ivermectin led to an inhibition percentage of 8.5%. This percentage increased by incubation with 100 μg/ml to reach 87%.

The weak response to ivermectin exposure at a concentration of 10 μg/ml could be attributed to the tolerance of *T. canis* larvae to the drug. Lespine *et al.*^36^ demonstrated that ivermectin is a known substrate of P-glycoproteins that efflux xenobiotics and drugs from cells. Jesudoss Chelladurai *et al.*^37^ tracked third-stage *T. canis* larvae incubated with ivermectin at a concentration of 10 μg/ml for 24 hours and recorded their motility using the WormLab software. The authors found that ivermectin alone did not inhibit larval movement at the specified concentration and duration of exposure. However, the combination of ivermectin with verapamil, a P-glycoprotein inhibitor, resulted in larval paralysis. Interestingly, *T. canis* P-glycoproteins are sensitive to macrocyclic lactones such as ivermectin and selamectin, but not to moxidectin ^38^. The *in vivo* results in the current work demonstrated the efficacy of ivermectin against early migrating *T. canis* larvae. However, Jesudoss Chelladurai *et al.*^37^ demonstrated P-glycoprotein mRNA expression, that leads to decrease the efficacy of the drug in both newly hatched migrating larvae and somatic larvae collected from infected mice after treatment with ivermectin.

The results of the larval migration inhibition assay in the present work correlated with the established mode of action of the tested drug that interferes with neurophysiology and neuromuscular coordination ^13^. The assay was used to evaluate the anthelmintic efficacy of drugs against nematode infective larvae ^25^^,^^31^ and was also used to identify resistance of several parasites to anthelmintics targeting the nervous system ^32^^,^^39^. The larval migration inhibition assay reduces the need for highly specialized technicians, requires less time and allows the screening of a larger number of third-stage larvae in each assay ^25^. Therefore, this test is an easy-to-use technique to measure the activity of compounds against third-stage larvae of *T. canis.*

The results of scanning electron microscopy study highlight that damage caused by ivermectin affects the cuticle, a critical structure in nematodes. Scanning electron microscopy examination of *T. canis* larvae incubated with 100 μg/ml ivermectin for 24 and 48 hours showed morphological changes in the cuticle. These changes intensified after 48 hours, with the complete disappearance of the transverse striations and the appearance of deep grooves with irregular edges. Similar abnormalities were observed in the larvae of *Trichinella spiralis* and *Brugia malayi* after incubation with ivermectin ^40^^,^^41^. The nematode cuticle has essential roles, as it functions as an exoskeleton and plays a crucial role in maintaining and defining the normal shape, facilitating movement, regulating permeability and defending the host immune system. Consequently, this structure is vital for the survival of the worm and represents a significant target for drug action ^42^.

In this study, larvae counts in the brain were analyzed to evaluate the efficacy of ivermectin when administered at different stages of *T. canis* migration. The results demonstrated significantly fewer larvae in the brains of ivermectin-treated mice at two and seven days post-infection compared to the control infected mice, with the most pronounced effect observed when treatment was administered at two days post-infection However, no significant reduction in larvae count was observed when ivermectin was given on day 15 post-infection The results of early administration of ivermectin, combined with its proven paralytic effect, suggest that the drug prevents a significant number of larvae from migrating to the brain and could lead to their eventual death, leading to a significant reduction in the total larvae count in the brain. Abo-Shehada and Herbert ^22^ reported that oral administration of ivermectin during the early phase of *T. canis* infection resulted in retention of larvae in the liver, where they subsequently died. Furthermore, administration of ivermectin on the seventh day post-infection was found to significantly reduce the number of *T. vitulorum* larvae recovered from various organs of rats ^43^.

After ingestion of infective *T. canis* eggs by paratenic hosts, including humans and mice, third-stage larvae hatch and penetrate the intestinal wall. By the second day, most larvae reach the liver, while on the third day, they migrate to the lungs, with a significant number remaining in the liver. Subsequently, the larvae migrate throughout the body and finally settle in the brain and muscles ^22^. *Toxocara canis* larvae exhibits pronounced neurotropism, showing a higher affinity for the brain compared to muscles ^44^^,^^45^. Therefore, the number of larvae present in the brain may indicate their ability to migrate through the blood and tissues, and the parasite load in the brain serves as an indicator of drug efficacy. Based on our results, it could be stated that early migratory larvae of *T. canis* are more susceptible to the effects of ivermectin than those already established in the brain.

Several studies have also shown that larval migration to the somatic musculature or the brain in mice allowed them to survive and be protected against several drugs ^22^^-^^24^. Although prenatal transmission of reactivated larvae in dogs has been prevented using ivermectin ^18^, non-reactivated somatic larvae are not eliminated by the drug or any other anthelmintic therapy ^19^. In mice infected with *T. spiralis,* early migrating larvae were more susceptible to ivermectin compared to later developmental stages ^40^.

The presence of numerous granulomas and dead larvae in the liver of mice treated with ivermectin early in the course of infection suggests that the immobilizing and toxic effects of the drug inhibit larval migration beyond the liver. Shehata *et al.*^43^ found that administering of ivermectin to rats at a dose of 0.2 mg/kg seven days post-infection reduced the number of *T. vitulorum* larvae in the liver.

Furthermore, in the present study, brain sections of mice treated from groups I and II revealed a lower number of larvae and reduced inflammatory reactions compared to the untreated control group. The reduction of inflammatory reactions could be attributed to the decrease in the number of larvae and the corresponding reduction in their excretory and secretory products, which serve as the main antigenic material stimulating immune responses ^46^. Moreover, ivermectin has been found to cause alterations in the excretory and secretory apparatus ^14^ that is the main source of these products release from *T. canis* larvae ^29^. This apparatus appears to be under neuromuscular control ^14^. Decreased secretion of these antigenic products may protect the host from brain damage and its sequelae ^47^.

The results also showed the ineffectiveness of ivermectin on larvae during the late migratory stage and after their settlement in the brain. This could explain the limited efficacy of ivermectin in the treatment of human toxocariasis ^48^ and in naturally infected dogs ^49^, particularly in cases where larvae have reached advanced migratory stages or have been localized in the brain. However, the combination of ivermectin with the P-glycoprotein inhibitor ^37^^)^ or with praziquantel ^49^ increases its efficacy. Fujii *et al.*^50^^)^ suggested that ivermectin could serve as an alternative treatment for toxocariasis, particularly in cases where albendazole is contraindicated due to hepatic impairment or other related problems. Ivermectin is also important in preventing infections. Somatic larvae residing in the tissues of bitches become reactivated during pregnancy and act as a reservoir of infection for up to three litters after a single exposure ^51^.

In summary, the current study demonstrated *in vitro* inhibition of *T. canis* larval migration after incubation with 100 μg/ml ivermectin. Incubation with the same dose for 24 and 48 hours resulted in cuticular damage, which was more pronounced after 48 hours, as observed by scanning electron microscopy. *In vivo* studies revealed that larvae in the early migratory phase are more susceptible to ivermectin, whereas resting hypobiotic somatic larvae in late infection exhibit tolerance to the drug. A possible avenue of research is to compare the expression of P-glycoproteins in larvae during early and late migratory stages.
